# Wealth, education and urban–rural inequality and maternal healthcare service usage in Malawi

**DOI:** 10.1136/bmjgh-2016-000085

**Published:** 2016-08-16

**Authors:** Sanni Yaya, Ghose Bishwajit, Vaibhav Shah

**Affiliations:** 1 Faculty of Social Sciences, School of International Development and Global Studies, University of Ottawa, Ottawa, Canada; 2 Institute of Nutrition and Food Science, University of Dhaka, Dhaka, Bangladesh; 3 Faculty of Health Sciences, Interdisciplinary School Health Sciences, University of Ottawa, Ottawa, Canada

## Abstract

**Background:**

Malawi is among the 5 sub-Saharan African countries presenting with very high maternal mortality rates, which remain a challenge. This study aims to examine the impact of wealth inequality and area of residence (urban vs rural) and education on selected indicators of maternal healthcare services (MHS) usage in Malawi.

**Methods:**

This study was based on data from the 5th round of Multiple Indicator Cluster Surveys (MICS) conducted in 2013–2014 in Malawi. Study participants were 7572 mothers aged between 15 and 49 years. The outcome variable was usage status of maternal health services of the following types: antenatal care, skilled delivery assistance and postpartum care. Univariate, bivariate and multivariate methods were used to describe the pattern of MHS usage in the sample population. Association between household wealth status, education as well as the type of residence, whether urban or rural, as independent variables and usage of MHS as dependent variables were analysed using the generalised estimating equations (GEE) method.

**Results:**

Mean age of the sample population was 26.88 (SD 6.68). Regarding the usage of MHS, 44.7% of women had at least 4 ANC visits, 87.8% used skilled delivery attendants and 82.2% of women had used postnatal care. Regarding the wealth index, about a quarter of the women were in the poorest wealth quintile (23.6%) while about 1/6 were in the highest wealth quintile (15%). Rate of usage for all 3 types of services was lowest among women belonging to the lowest wealth quintile. In terms of education, only 1/5 completed their secondary or a higher degree (20.1%) and nearly 1/10 of the population lives in urban areas (11.4%) whereas the remaining majority live in rural areas (88.6%). The rates of usage of MHS, although reasonable on an overall basis, were consistently lower in women with lower education and those residing in rural areas.

**Conclusions:**

Maternal health service usage in Malawi appears to be reasonable, yet the high maternal mortality rate is disturbing and calls for analysing factors hindering the achievement of maternal health-related Sustainable Development Goals (SDGs). The findings of this study underscore the need to minimise the wealth inequality, urban–rural divide and the low level of education among mothers to improve the usage of MHS. An equity-based policy approach considering the sociodemographic inequity in terms of wealth index, education and urban–rural divide might prove beneficial in further improving the MHS usage, as well as addressing the possible issues of quality gaps in MHS, which might be beneficial towards reducing maternal mortality. It should be noted that the study of quality gaps in MHS is beyond the scope of this paper and calls for further research in this arena.

**Key questions**

**What is already known about this topic?**

- In developing countries, underuse of maternal healthcare services is attributed to an array of supply and demand factors, social structure and health beliefs.
- Among various socioeconomic factors, maternal education level and economic status are the most important determinants of usage of health services.

**What are the new findings?**

- Wealth status, education and area of residence (urban vs rural) have a significant impact on uptake of all three types of maternal health services in the study population.
- Compared with the women in the poorest wealth quintile, those in the higher quintile have significantly higher odds of receiving at least four antenatal care visits, skilled birth attendance and postnatal care.
- Comparatively, women with relatively higher education had higher odds of receiving at least four antenatal care visits, skilled birth attendance and postnatal care.
- Women in rural areas were less likely to receive four antenatal visits, skilled birth attendance and postnatal care.
- Antenatal care usage is particularly lower in Malawi and more focus should be directed at improving antenatal visits as per the WHO standards.

**Recommendations for policy**

- Provision of quality healthcare by increasing education and reducing area (urban vs rural) and wealth inequality should be a top public health priority in the Sustainable Development Goals (SDGs).
- Since the healthcare system is fraught with a range of funding and logistical issues, more nuanced cooperation between local and international development organisations is needed to successfully achieve the maternal health-related targets in the country.
- The Government of Malawi should invest more in education and infrastructural development to begin removing the structural causes of non-use of maternal healthcare services.

## Introduction

Despite constant efforts by the global community to reduce the burden of death arising from pregnancy and childbirth, maternal mortality (maternal deaths/100 000 live births) still remains a serious issue, affecting about 800 lives a day.^1^ Pregnancy/childbirth is arguably the most anticipated event in the life of a woman, yet complications during this period (from pregnancy to delivery) constitute the leading cause of death among women aged between 15 and 49 years.^2^ The Millennium Development Goal 5 (MDG 5) was dedicated to the goal of reducing, between 1990 and 2015, the maternal mortality ratio (MMR) by three-quarter.^3^

Although some countries have shown promising outcomes in terms of reduction in MMR, many are still behind track and continue to face maternal mortality as a major population health challenge. Statistics reveal that practically all-maternal deaths (99%) occur in the low and middle income countries (LMICs), among which Africa alone accounts for over 50% of the deaths.^1^ According to the reports by the United Nations Population Fund (UNDP), UNICEF and WHO, maternal mortality is as high as 1 in 11 in Eastern Africa compared with 1 in 3500 in North America and 1 in 4000 in Western Europe.^4^ The most important causes of maternal mortality in developing countries are unsafe abortion, haemorrhage, eclampsia and obstructed labour as they together account for nearly two-third of total maternal mortality globally.^1^ ^3^ ^5^ A growing consensus suggests that a vast majority of these deaths are actually preventable by adopting the necessary precautions provisioned through basic maternal healthcare services (MHS).^6^

The basic components of MHS: (1) during pregnancy (antenatal care), (2) during the intrapartum period (labour and delivery) and (3) during the postpartum period, postdelivery has been proposed as a key strategy to combat maternal mortality in resource-poor countries such as Malawi. In developed countries, nearly all women (98%) receive antenatal care (ANC) and deliver under the supervision of skilled health professionals (94%).^7^ In LMICs, on the other hand, about half of the women are deprived of ANC services^8^ and more than half of all births take place, outside institutional settings, mostly in unhygienic and unsafe conditions.^9^ ^10^ The WHO recommends at least four antenatal visits for all pregnant women. However, almost half of the pregnant women worldwide do not receive this level of care, which is more common in LMICs. Poor attendance of ANC is associated with increased rates of low birthweight babies and neonatal deaths. Again, postpartum care is a crucial need for the survival of the mother and the newborns, as most maternal and infant deaths occur during this period. The WHO recommends that mothers and newborns receive initial postnatal care (PNC) within the first 24 hours after delivery and a minimum of three additional PNC visits within 48–72 hours, 7–14 days and 6 weeks after delivery.^11^ Despite the strong recommendation of the WHO in this context, this is the most neglected period for the provision of quality care.^12^ Unfortunately, a great majority of the women in LMICs remain deprived of the basic MHS due to various socioeconomic^13^ and cultural constraints.^14^

Again, at the advocacy level, these issues are treated with a reductionist approach, although with a purpose only to relegate the complexities of implementation, given the diverse and intricate case-specific realities.^15^ In one of the studies in Tanzania, for example, despite higher antenatal care coverage and a positive notion about antenatal care on the part of women, there were significant gaps in antenatal care quality, based on factors such as avoiding repeated visits to the clinic, lack of money or concerns about caesarean deliveries.^16^ This catches the moot point of approaching indicators of maternal health like skilled birth attendance; antenatal care coverage and postnatal care with caution. Reasonable indicators may not necessarily mean reasonable maternal health. For example, the fuller potential of antenatal care strategy in reducing maternal mortality can be accomplished by providing what is called a ‘focused antenatal package’, including identifying possible obstetric complications and planning in advance for the emergency obstetric care possibilities and realities in terms of geographical location and service availabilities among others. A study in Uganda found that emergency obstetric care is a priority in reducing maternal mortality.^17^ Yet these aspects of MHS remain neglected.

The topic of socioeconomic inequality has received growing research attention in the domain of population health. Numerous studies have shown that the economically disadvantaged sections of the society are also the ones that suffer the worst health conditions. However, the association between economic inequality and MHS usage is less widely studied. Intuitively, economic constraints are a strong limiting factor for the accessibility and affordability of healthcare services for mothers from poor households. Women from well-off families are more likely to be able to pay for the costs associated with healthcare visits, medications and transportation.

Evidence from a Multiple Indicator Cluster Survey (MICS; 2007) study in Vanuatu found that mothers in the highest wealth status were, respectively, 5.50 and 2.12 times more likely to be assisted by skilled birth assistance (SBA) and have institutional deliveries.^14^ One Malawian study based on Demographic and Health Surveys (DHS 1992, 2000 and 2004) concluded that non-poor who suffered less frequently from selected diseases (including selected MHS) received more of the treatment/interventions, compared with the poorer counterparts, who with a greater proportion of disease burden get less of the interventions.^18^ Experience from other sub-Saharan nations also reveal similar situations.^19^ ^20^ Inequality in MHS can greatly thwart progress towards maternal mortality-related MDGs and thus impede national progress owing to the direct and indirect losses arising from poor maternal and child health. It should also be noted that wealth inequality cannot be isolated from the compounding effects of other factors like education and the place of residence, that is, urban versus rural. With an aim to understand maternal healthcare seeking behaviour in relation to wealth inequality, education level and differences between urban and rural residence in Malawi, we conducted this study by analysing the most recent MICS data and estimated the rate of MHS usage and how usage status varies across selected variables.

## Methods

### About the survey and study population

The MICS programme has the recognition of being the most comprehensive and reliable source of data on maternal and child health issues in developing countries. Operating in technical cooperation with UNICEF, the programme encompasses 108 countries and has completed over 280 surveys since its inception in 1995. The data sets serve crucial tools for monitoring progress towards MDGs and has become a vital component for evidence-based public health and social policymaking across countries. Data for the present study on Malawi was obtained from the fifth round of the survey (MICS 5), which was conducted in Malawi, from November 2013 to April 2014. The survey (Malawi MDG Endline Survey 2014) included information on various indicators of MDGs and other key socioeconomic and demographic variables and was carried out with an aim to measure progress towards MDGs and other development programmes in the country.^21^ The survey employed a multistage cluster-sampling strategy to select a sample population in 27 districts. In total, 24 230 women aged between 15 and 49 years were interviewed, with a response rate of 95.3%. However, an inclusion criterion for this study was the birth of a child in the past 2 years.

### Selection of variables

The outcome variables of interest were antenatal care, skilled birth attendance and postnatal care. Wealth status, type of residence, that is, urban versus rural and education were the independent variables of interest. MHS included three basic components and was categorised as yes/no in SPSS, the statistical software package used for analysing the data. The component of MHS included the following:

1. During pregnancy (ANC): As per the recommendation by World Health Organization, ANC was defined as having at least four visits to a qualified healthcare provider in their pregnancy.
2. During the intrapartum period (SBA): This was determined by the usage of SBA during delivery.
3. During the postpartum period (PNC): Whether or not mothers underwent a health check-up after delivery. The study considered a postnatal check-up within 48 hours after birth as a potential maternal healthcare indicator as per the WHO.

Wealth index: Household wealth status is representative of an individual's affordability of expenses arising from healthcare needs. MICS programmes employ wealth index as a proxy indicator for household wealth status. The process involves assigning wealth scores, which is performed by principal components analysis, based on a selected range of household assets, for example, number of household members, floor, wall and roof material; type of cooking fuel; access to potable water and sanitation, ownership of radio, TV, refrigerator, motorcycle and others. Based on their weighted wealth scores, households fall into five wealth quintiles ranging from poorest to richest (poorest, poorer, middle, richer, richest). Measurement of wealth index is explained in detail elsewhere.^21^

Education: Education of the mother was categorised as those having education up to primary school and those having education of secondary school or higher.

Type of residence: This variable included the categories of urban and rural residences.

### Covariates

Defining explanatory variables: Selected socioeconomic and demographic variables such as mother's age at birth, attended school: yes/no; religious faith; wealth quintile (poorest, poorer, middle, richer, richest); type of residence, that is, urban versus rural and geographical region of residence, that is, Northern, Central or Southern, were used as covariates. Three outcome variables for MHS included in the study were antenatal care, skilled birth attendance and PNC for mothers.

### Data analysis

The sociodemographic characteristics of participants were analysed by descriptive statistics. Cross-tabulation was performed, and χ^2^ bivariate tests were used to check for statistical significance with MHS usage status and as a guide to the explanatory variables which are to be included in the multivariate analysis. All the covariates were entered as categorical variables. Variables that were found to have significant association from the χ^2^ test in cross-tabulation were entered into the regression model. Given the clustered nature of the survey, we used generalised estimating equations (GEE) for regression analysis. The aim of the final analysis was to adjust for potential confounders and calculate the ORs to assess the likelihood of using MHS. A p Value of <0.05 (two-tailed) was considered statistically significant for all associations. Data analysis was performed using SPSS V.24 for Windows (SPSS, Chicago, Illinois, USA).

### Ethical approval

This research was based on secondary data available in the public domain by the courtesy of the MICS programme of UNICEF and hence was not subject to ethical approval.

## Results

### Sociodemographic characteristics

**Figure 1:**
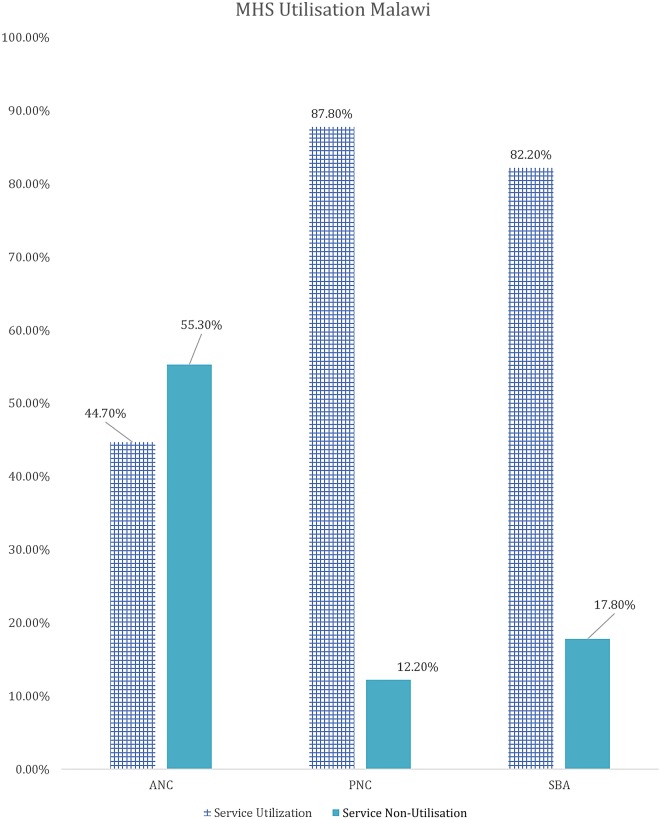
Use of MHS by type in Malawi. MICS 2013–2014. ANC, antenatal care; MHS, maternal healthcare service; MICS, Multiple Indicator Cluster Survey; PNC, postnatal care; SBA, skilled birth assistance.

**Figure 2:**
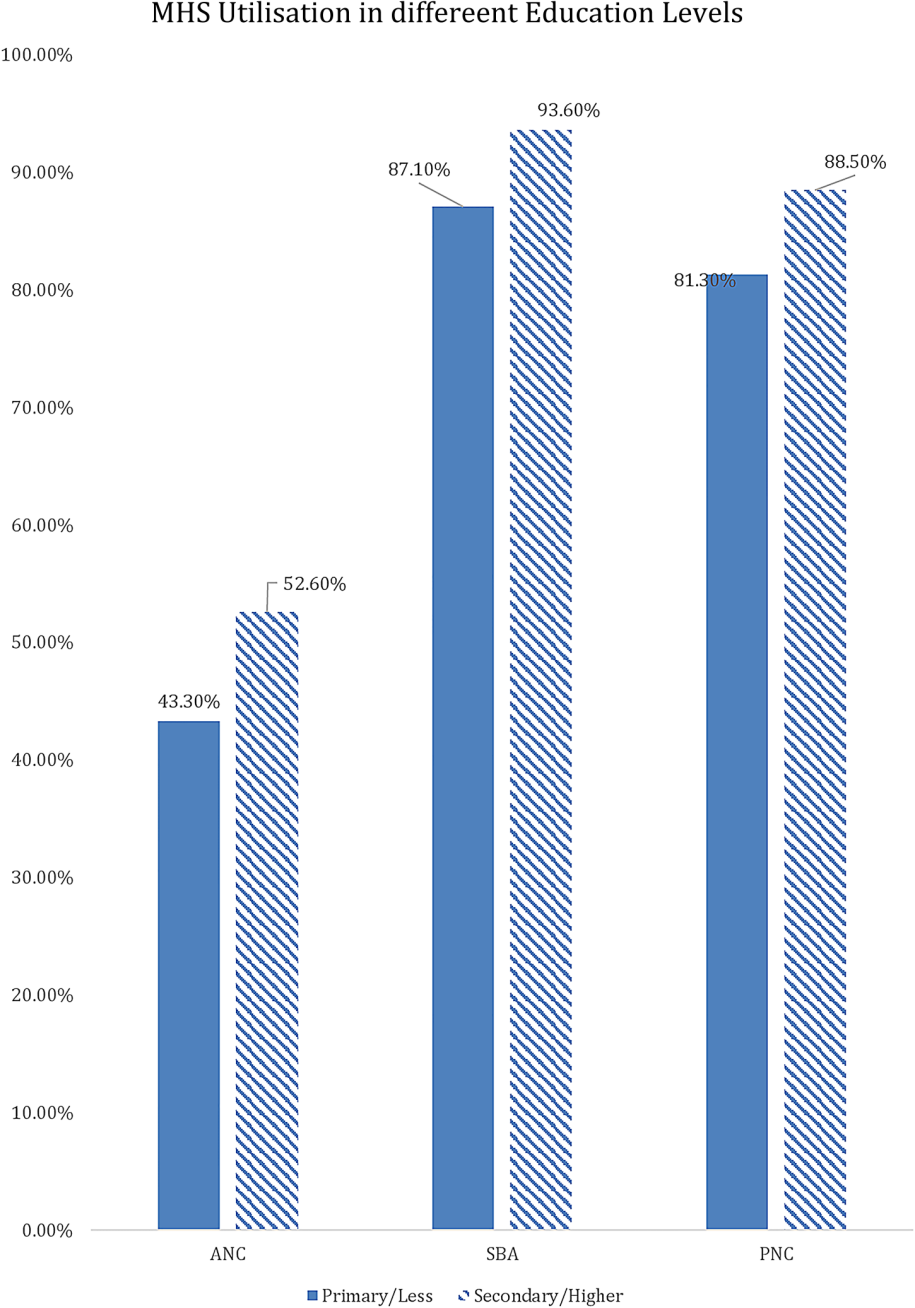
Utilisation rate of MHS at different education levels in Malawi. MICS 2013–2014. ANC, antenatal care; MHS, maternal healthcare service; MICS, Multiple Indicator Cluster Survey; PNC, postnatal care; SBA, skilled birth assistance.

Table 1 shows the results of the descriptive analysis (frequencies and percentages) on the sociodemographic characteristics of the sample population. In total, 7572 women were included in the study with an average age of 26.88 (±6.68) years. A high number of pregnancies (46.5%) were noted in the high-risk age range, either between 15 and 19 years or above 30 years. It should be noted as per Donoso *et al*^22^ that 20–29 years is the age range with a lesser general reproductive risk. About 1 in 10 reported ‘yes’ to the question ‘ever attended school’ (88.8%). Nearly one-fifth of the population belonged to the Christian faith. The population with Muslim faith was 14.2%, and 3.8% and 0.5% belonged to either no religion or other religion, respectively. In terms of education, only 20.1% had secondary education or higher and 79.9% had education up to primary school. Wealth status quintiles were distributed in the study sample, with 23.6%, 22.7% and 21.3% belonging to the poorest, poor and middle quintiles, respectively, whereas 17.3% and 15% belonged to the rich and richest quintiles of the study sample, respectively. The majority (88.8%) of the study sample belonged to the rural areas, whereas geographical distribution of the study sample closely matched the census 2010 data, with 17.2% belonging to the Northern region, 33.9% belonging to the Central region and 49% belonging to the Southern region. Rates of use of MHS by type, usage of MHS at different education levels, wealth groups and areas (urban vs rural) are presented in figures 1–4 below. As seen in figure 1, only 44.70% of women use antenatal care (ANC) services as per the WHO defined criteria of at least four ANC visits during pregnancy. The rates of use of PNC and SBA are above 80%. Similarly, figure 2 shows the usage of MHS, ANC, PNC and SBA at different education levels. Figure 3 shows usage of MHS in different wealth quintiles and figure 4 shows the usage of MHS in different areas.

**Table 1:** Cross-tabulation results with covariates

Covariates; n=7572	n%	ANC>4 times	SBA	PNC
Age				
Low risk group	4052 (53.50%)	1735 (43.90%)	3566 (89%)	3282 (82.50%)
High risk group	3520 (46.50%)	1558 (47.30%)	3011 (86.40%)	2832 (81.80%)
p Value		NS	p<0.001	NS
Education				
Primary/less	5372 (79.90%)	5219 (43.30%)	4635(87.10%)	4288 (81.30%)
Secondary/higher	1351 (20.10%)	700 (52.60%)	1254 (93.60%)	1183 (88.50%)
p Value		p<0.0001	p<0.0001	p<0.0001
Religion				
Christian	6176 (81.60%)	2714 (45.10%)	5371 (87.80%)	5031 (82.80%)
Muslim	1072 (14.20%)	450 (43.60%)	935 (88.30%)	838 (79.60%)
No religion	285 (3.80%)	115 (41.10%)	235 (83.60%)	215 (78.50%)
Other	39 (0.50%)	14 (36.80%)	36 (92.30%)	30 (78.90%)
p Value		NS	NS	p=0.012
Wealth index				
Poorest	1789 (23.60%)	721 (41.40%)	1506 (84.80%)	1379 (78.21)
Second	1720 (22.70%)	711 (42.60%)	1468 (86.30%)	1355 (80.60%)
Middle	1616 (21.30%)	684 (43.3%)	1408 (87.90%)	1310 (82.30%)
Fourth	1312 (17.30%)	587 (46%)	1148 (88.70%)	1082 (84.10%)
Richest	1135 (15%)	590 (53.60%)	1047 (93.40%)	988 (88.50%)
p Value		p<0.0001	p<0.0001	p<0.0001
Region				
Northern	1299 (17.20%)	542 (42.30%)	1154 (89.50%)	1118 (87.50%)
Central	2566 (33.9%)	1117 (44.6%)	2204 (86.80%)	2110 (83.60%)
Southern	3707 (49%)	1634 (45.70%)	3219 (87.90%)	2886 (79.40%)
p Value		NS	p=0.025	p<0.0001
Area				
Urban	866 (11.40%)	458 (54.10%)	800 (93.20%)	758 (88.60%)
Rural	6706 (88.60%)	2835 (43.50%)	5277 (87.10%)	5356 (81.30%)
p Value		p<0.0001	p<0.0001	p<0.0001

The p denotes level of significance estimated from the χ^2^ test.

ANC, antenatal care; NS, not significant; PNC, postnatal care; SBA, skilled birth assistance.

**Figure 3:**
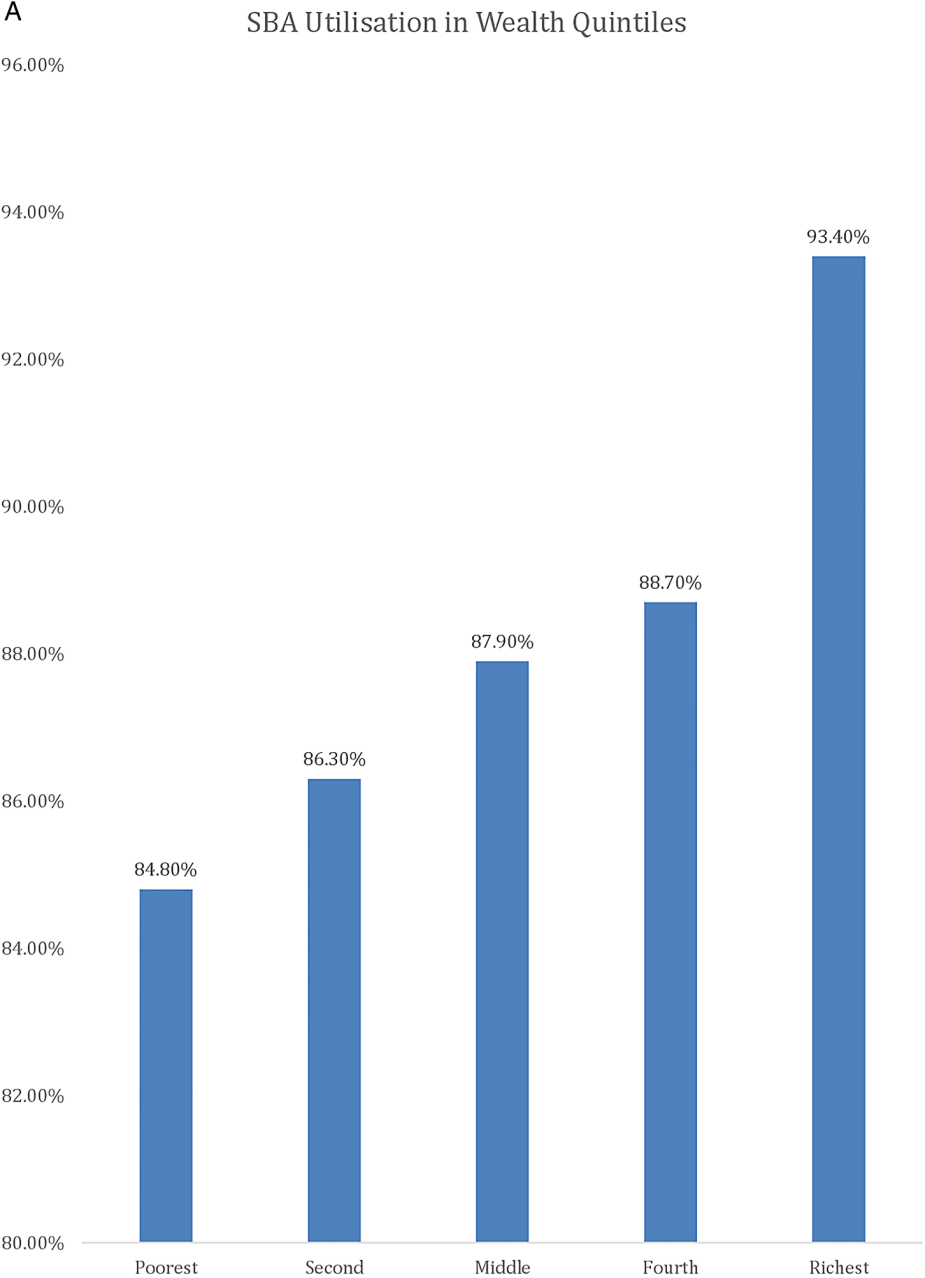
Utilisation rate of MHS in different wealth groups in Malawi. MICS 2013–2014. ANC, antenatal care; MHS, maternal healthcare service; MICS, Multiple Indicator Cluster Survey; PNC, postnatal care; SBA, skilled birth assistance.

**Figure 3:**
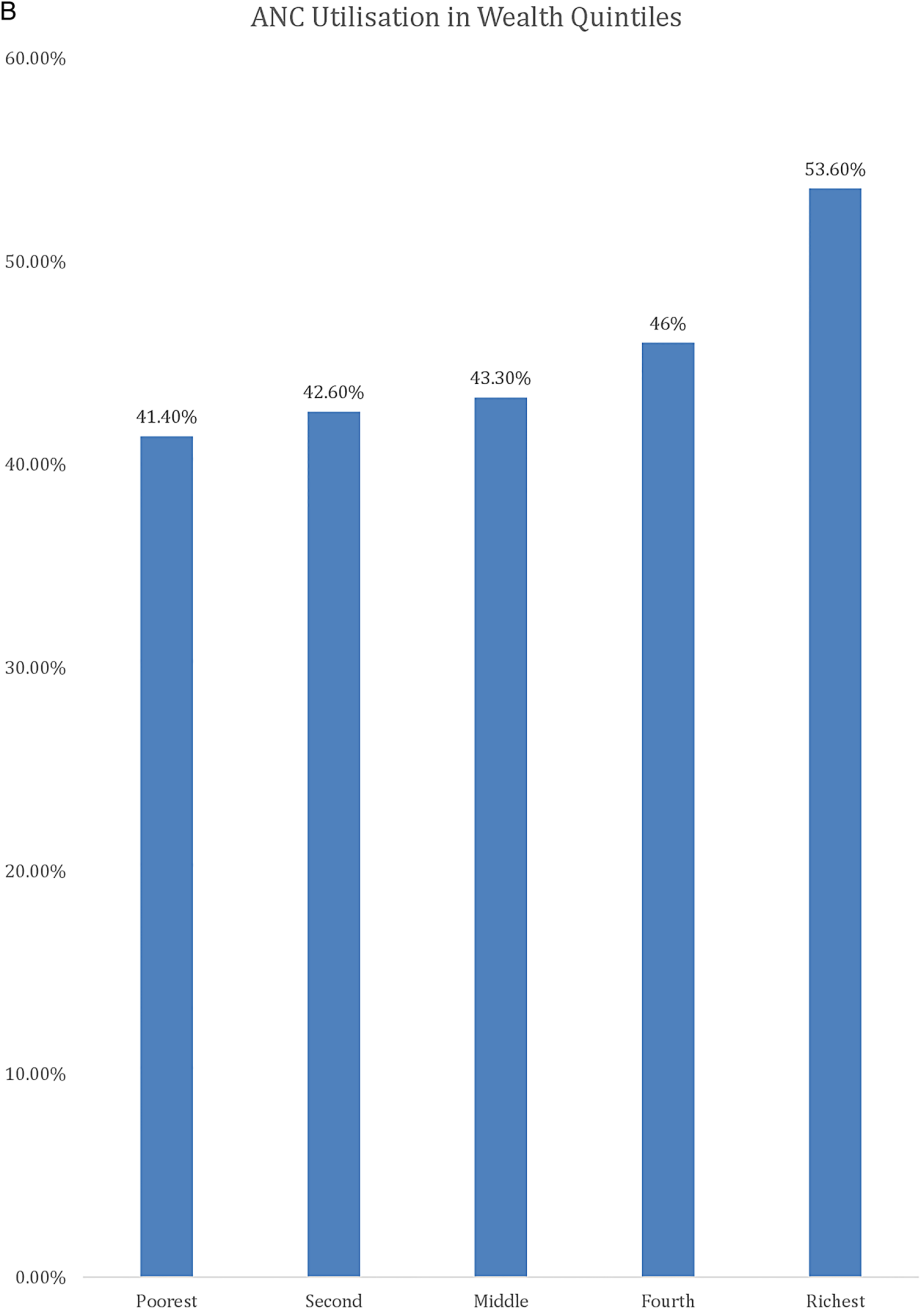
Continued

**Figure 3:**
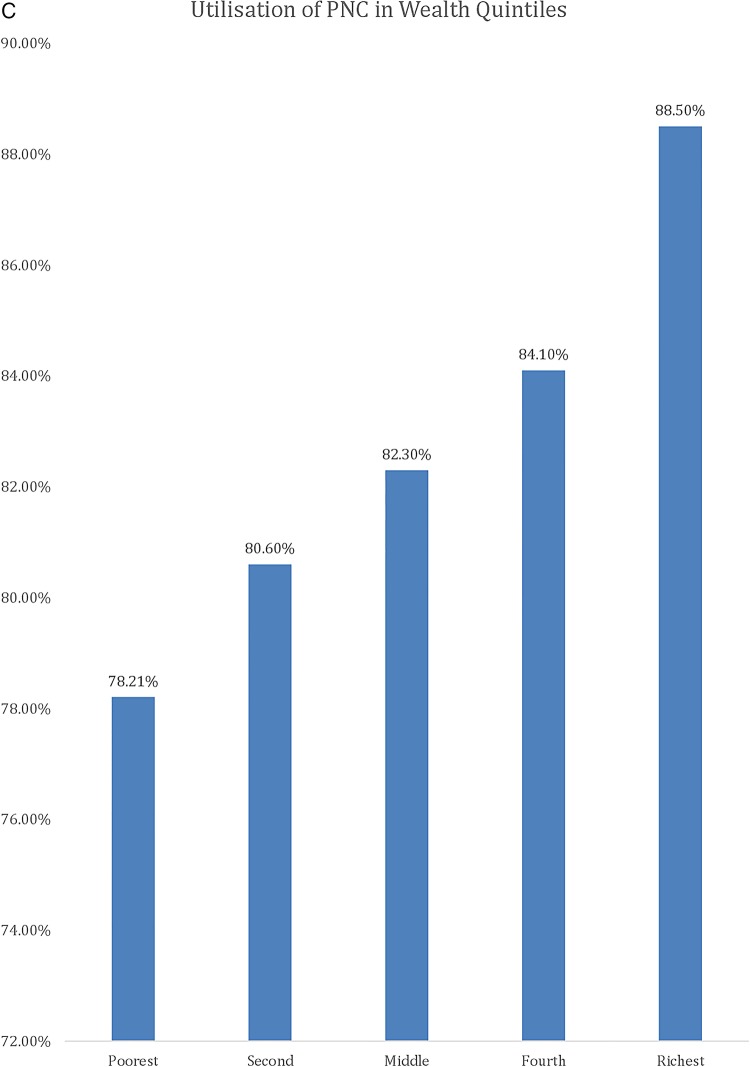
Continued

**Figure 4:**
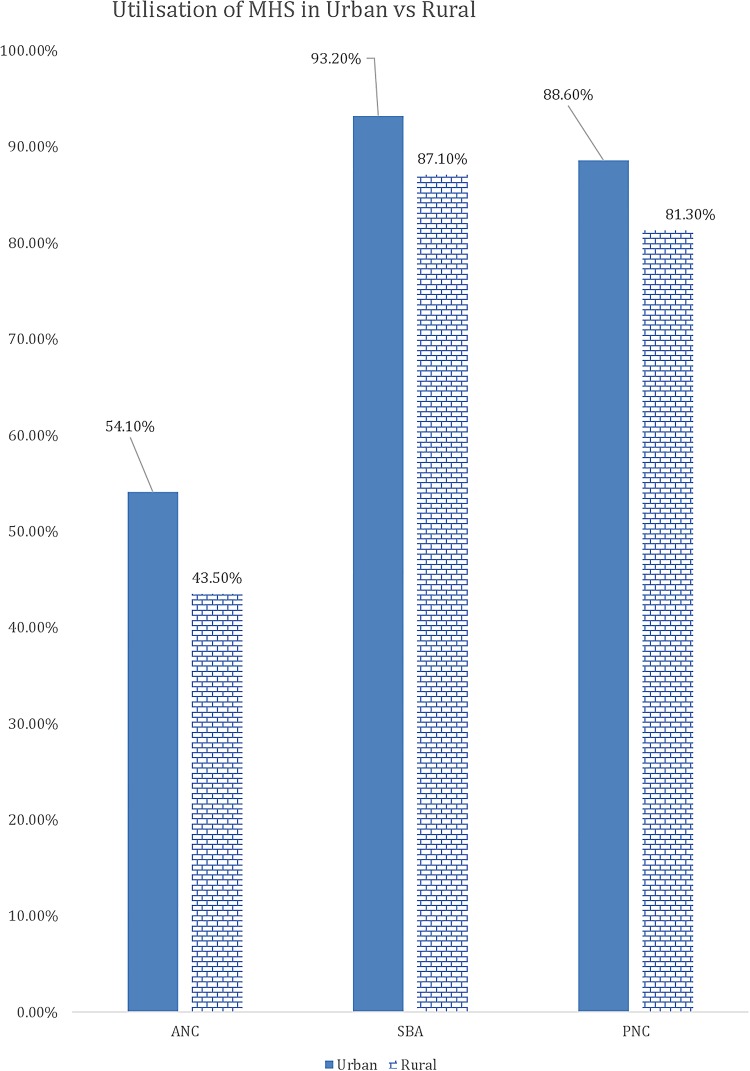
Utilisation rate of MHS in different areas in Malawi. MICS 2013–2014. ANC, antenatal care; MHS, maternal healthcare service; MICS, Multiple Indicator Cluster Survey; PNC, postnatal care; SBA, skilled birth assistance.

As per the χ^2^ tests, education, wealth index quintiles and area (urban vs rural) were significantly associated with the usage status of all the three types of MHS. Detailed cross-tabulation results are as displayed in table 1 below.

Table 2 shows the usage of additional health services generally prescribed for pregnant mothers. Well over four-fifth of the women had their blood pressure measured and a little less than one-third had their urine sample tested. A majority of the women (93.7%) had their blood sample taken during pregnancy.

**Table 2:** Additional health services during pregnancy

Tests	Percentage of women who reported yes
Any tetanus toxoid injection during pregnancy	83.40
Blood pressure measured during pregnancy	87.70
Blood sample taken during pregnancy	93.70
Urine sample taken during pregnancy	31.30

### Multivariate analysis

Table 3 shows the ORs with 95% CIs obtained from the multiple logistic regression for usage status of MHS. Logistic regression was performed to ascertain the effects of wealth index, education, area of residence, that is, urban versus rural, religion and region on the likelihood that participants have used ANC. The logistic regression model was statistically significant, χ^2^ (5)=68.204, p<0.0001. The model explained 1.4% (Nagelkerke R^2^) of the variance in ANC and correctly classified 56.3% of cases. Sensitivity was 20.3%, specificity was 85.9%, positive predictive value was 54.3% and negative predictive value was 56.63%. Of the five predictor variables, only four were statistically significant: wealth index, education, area and region. Moving from poorest to richest, a one-unit increase in the wealth index quintile had 1.063 times higher odds of having used ANC services as per the WHO standards. Similarly, moving from primary or lower education to secondary or higher education had 1.3 times higher odds of having used ANC services at the given standards. A unit increase in area, that is, moving from urban to rural, decreased the likelihood of using ANC services and, in terms of region, moving from the Northern to Southern region increased the likelihood of using ANC services. The results of logistic regression are as shown in table 3 below.

**Table 3:** Results of logistic regression model showing OR and CI, sensitivity, specificity and PPV and NPV for respective models, for usage of different types of MHS among women in Malawi, 2013–2014

Variable	OR	Sensitivity (%)	Specificity (%)	PPV (%)	NPV (%)
SBA					
Wealth index	1.10 (1.03 to 1.17)	100	0	88.40	0
Education	1.79 (1.40 to 2.29)				
Area	0.68 (0.50 to 0.93)				
Religion	1.00 (0.87 to 1.16)				
Region	1.02 (0.92 to 1.13)				
PNC					
Wealth index	1.08 (1.02 to 1.14)	100	0	82.70	0
Education	1.44 (1.19 to 1.75)				
Area	0.71 (0.55 to 0.91)				
Religion	0.94 (0.83 to 1.06)				
Region	1.02 (0.92 to 1.13)				
ANC					
Wealth index	1.06 (1.02 to 1.10)	20	86	54.30	56.60
Education	1.30 (1.14 to 1.48)				
Area	0.81 (0.69 to 0.96)				
Religion	0.99 (0.89 to 1.09)				
Region	1.10 (1.03 to 1.18)				

ANC, antenatal care; NPV, negative predictive value; PNC, postnatal care; PPV, positive predictive value; SBA, skilled birth assistance.

## Discussion

On the basis of the MICS wave 5 data, this study attempts to demonstrate the impact of wealth inequality, education and area of residence on selected indicators of MHS usage in Malawi. Despite a considerable drop in MMR at the global stage during the past few decades (44% between 1990 and 2015), progress has been lowest in sub-Saharan Arica (SSA) as the countries continue to share a disproportionate burden of maternal and neonatal mortalities. According to the 2015 MDG progress report, SSA accounts for about two-third of maternal and half of neonatal mortality globally.^23^

The same report classified Malawi as having a very high MMR in 2015 with about 634 deaths per 100 000 live births. Several studies have attempted to explore the root causes of high MMR in Malawi. While a growing body of literature is documenting the impact of inequality, education and area of residence, that is, urban versus rural, in maternal health, quality evidence is lacking for countries in SSA. Previous experience from developing regions in Asia and Africa reveals a positive association between MHS uptake and wealth inequality.^19^ ^24^ ^25^ Studies have also indicated the impact of education and the area of residence, that is, urban versus rural, on maternal health services usage.^26^

In this study, we sought to investigate how household wealth inequality, area (urban vs rural) and education affect the usage status of MHS among Malawian women. Our finding showed that wealth status had a significant impact on uptake of all three types of MHS in the study population. Compared with the women in the poorest wealth quintile, those in the higher quintile have significantly higher odds of receiving at least four ANC visits, skilled birth attendance and attending PNC. Our results are consistent with past findings. A previous study by analysing DHS data for the years between 1990 and 1998 in 45 developing countries showed that the use of skilled assistance at delivery and antenatal care is 80% or higher for the richest quintile.^27^

Another DHS study in 56 countries during 1990–2002 found that women in the richest quintile were nearly five times more likely to experience skilled assistance at delivery than the poorest.^28^ This finding indicates that wealth inequality is a limiting factor for MHS usage in Malawi. Past evidences suggest that addressing wealth inequalities in MHS usage is essential for achieving the maternal health-related MDGs.^11^

Financial barriers to usage of facility-based care are prohibitive among the poor, even where the actual care is free of charge. Some countries in the SSA are implementing policies to lower/exempt direct out-of-pocket (OOP) costs^29^ to promote maternal health in the region. Direct OOP costs associated with maternity care include all formal, official fees charged for delivery care, bed stay, and required drugs and supplies. In addition to direct financial expenditures, there may be additional indirect costs of care seeking, such as lost wages or earnings. Such costs are difficult to measure as they vary according to income and employment status, and may be subject to seasonal variation as well. Indirect costs of care seeking can exceed direct OOP costs. Owing to system inefficiency and poor accountability and transparency, unofficial fees were on average 12 times higher than official fees.^30^ National health policymaking should take into account the direct as well as indirect expenditures to promote MHS uptake among the disadvantaged sections of the society.

In Malawi, healthcare financing is faced with serious constraints and is highly dependent on external sources of financing. Budgetary failure (Abuja Declaration of 15% of the national budget), decreasing share of private sources in healthcare expenditure and rising health expenditure (US$12 in 1998/1999 to US$25 in 2005/2006) are concerns for healthcare financing in the country.^31^ More than three quarters of the population live below poverty line. Hence despite reduction in out-of-pocket payments, this still remains worth consideration. Poor households usually spend a large share of income for food and any amount of spending can be competitive for household food availability and education of children. Thus, the burden of maternal healthcare is unlikely to be affordable especially for poor households.

Similarly, those residing in rural areas are significantly less likely to use maternal health services as seen by logistic regression results. Education was another variable, which had a significant impact on utilisation of all three types of services. Those with no education or up to primary school level had lower odds for using all the three types of MHS compared with those with secondary or higher education. Given the relatively higher rates of usage of all three types of MHS, the impact of area and education become even more relevant. It raises a moot point about the quality of MHS, which might be subject to the impact of area and education. For example, a study from Tanzania noted that those in a rural set-up face barriers of transportation and reaching the health facility to receive appropriate antenatal care.^32^ Area of residence impacts the quality of MHS services through standards of care for antenatal visits, timing of postpartum care and identification of intra-partum risk factors, as found from one of the studies in rural India.^33^

Another qualitative study from Malawi has shown that there are factors apart from maternal health services usage, like lack of appropriate resources for maternal health services, overloaded staff, etc. A study from Sudan also revealed a substantial impact of education and area of residence on the quality of maternal health services received as well as the rates of maternal health services.^34^ Another study from rural Tanzania revealed the overarching influence of the rural set-up on the perception of postpartum complication and quality of health services received.^16^ It should be noted, however, that this study is related to the impact of area of residence, education and wealth inequity on the usage of maternal health services and the point about quality of maternal health services calls for further research. The discussion does, however, underscore the influence of factors beyond mere utilisation rates of maternal health services and emphasises the import of focusing on reducing maternal mortality versus improving indicators of maternal health.

### Conclusion

The finding of this study reveals reasonable rates of usage of MHS. The rates of usage are significantly impacted by the differences in education, area of residence and wealth inequalities. Barriers to maternal health care due to socioeconomic and cultural factors are well recognised in the country, which necessitates special intervention programs that directly benefit the poor, particularly in most underdeveloped areas. The focus should also be on increasing women's education above secondary/higher levels. Despite reasonable rates of MHS usage, the maternal mortality rates continue to remain high. Therefore, provision of quality healthcare by increasing education and reducing wealth inequality as well as reducing the urban–rural divide should be a top public health priority in the Sustainable Development Goals (SDGs). Since the healthcare system is fraught with a range of funding and logistical issues, more nuanced cooperation between local and international development organisations is needed to successfully achieve the maternal health-related targets in the country.

### Limitations of the study

As an observational study, the findings do not indicate a cause–effect relationship between wealth inequality, education and area of residence with usage of MHS. The survey also relied on participants' ability to correctly recall the timing and frequency of the services they availed. So there is a strong possibility of recall error and under-reporting by the participants.
